# Roles of interacting stress-related genes in lifespan regulation: insights for translating experimental findings to humans

**Published:** 2021-10-19

**Authors:** Anatoliy I. Yashin, Deqing Wu, Konstantin Arbeev, Arseniy P. Yashkin, Igor Akushevich, Olivia Bagley, Matt Duan, Svetlana Ukraintseva

**Affiliations:** Biodemography of Aging Research Unit, Duke University, Durham, NC 27705, USA

**Keywords:** Integrated stress response, amino acids starvation, health and lifespan, *GCN2/EIF2AK4* and *CHOP/DDI3T* genes, GxG interactions

## Abstract

**Aim:**

Experimental studies provided numerous evidence that caloric/dietary restriction may improve health and increase the lifespan of laboratory animals, and that the interplay among molecules that sense cellular stress signals and those regulating cell survival can play a crucial role in cell response to nutritional stressors. However, it is unclear whether the interplay among corresponding genes also plays a role in human health and lifespan.

**Methods:**

Literature about roles of cellular stressors have been reviewed, such as amino acid deprivation, and the integrated stress response (ISR) pathway in health and aging. Single nucleotide polymorphisms (SNPs) in two candidate genes (*GCN2/EIF2AK4* and *CHOP/DDIT3*) that are closely involved in the cellular stress response to amino acid starvation, have been selected using information from experimental studies. Associations of these SNPs and their interactions with human survival in the Health and Retirement Study data have been estimated. The impact of collective associations of multiple interacting SNP pairs on survival has been evaluated, using a recently developed composite index: the *SNP-specific Interaction Polygenic Risk Score* (SIPRS).

**Results:**

Significant interactions have been found between SNPs from *GCN2/EIF2AK4* and *CHOP/DDI3T* genes that were associated with survival 85+ compared to survival between ages 75 and 85 in the total sample (males and females combined) and in females only. This may reflect sex differences in genetic regulation of the human lifespan. Highly statistically significant associations of SIPRS [constructed for the rs16970024 (GCN2/EIF2AK4) and rs697221 (CHOP/DDIT3)] with survival in both sexes also been found in this study.

**Conclusion:**

Identifying associations of the genetic interactions with human survival is an important step in translating the knowledge from experimental to human aging research. Significant associations of multiple SNPxSNP interactions in ISR genes with survival to the oldest old age that have been found in this study, can help uncover mechanisms of multifactorial regulation of human lifespan and its heterogeneity.

## INTRODUCTION

### The multifactorial nature of aging, health, and lifespan-related traits is broadly recognized but understudied

It is generally acknowledged that human lifespan, aging, and age-associated health disorders are multifactorial traits resulting from the complex interplay among numerous genetic and non-genetic factors. Observed correlations between biomarkers of biological aging and age-associated diseases indicate a possibility of improving health and increasing lifespan through deceleration of the aging-related processes in the body. A better understanding of the mechanisms of multifactorial regulation of respective traits could substantially facilitate the realization of this idea.

Numerous experiments using animal models were performed to improve such understanding. Surprisingly, a number of studies revealed that mutations in just one gene in *C. elegans* could substantially increase the lifespan of laboratory animals (reviewed in^[1]^). E.g., Johnson *et al.*^[2,3]^ identified the long-lived mutant of *C. elegans* called *age*-1. Later, other mutants with substantially longer survival compared to wild animals were detected^[1,4,5]^. Note that the effects of such mutations on lifespan in other species are much less pronounced.

Many other experimental studies discovered that better health and longevity could be achieved in animals from different species exposed to caloric/dietary restriction (CR/DR) (reviewed in^[6–9]^). Further research showed that separate components of the diet, including carbohydrates^[10,11]^, lipids^[12]^, proteins^[13–16]^, vitamins^[17]^, minerals^[18]^, fiber^[19]^, and water, can influence aging and lifespan through different albeit often interacting genetic regulatory mechanisms. Experiments with different mutants of *C. elegans*^[20]^ and different strains of mice^[21]^ overall suggested that the effects of CR/DR on lifespan can have a strong genetic component. The genetic mechanisms involved in lifespan regulation in response to amino acid deprivation were linked to genes from the mTOR signaling pathway^[22,23]^. It has also been shown that amino acid deprivation may influence lifespan by activating interplay among genes from the integrated stress response (ISR) pathway with the *GCN2/EIF2AK4* gene serving as a sensor of such stress signals and the *CHOP/DDIT3* gene serving as a regulator of cell’s fate deciding between autophagy, cell cycle arrest, or apoptosis^[24–26]^. Apoptosis is a cellular choice when stress response cannot restore the normal cell’s functioning.

The genetic epidemiological genome-wide and candidate genes single locus studies of human aging, health, and longevity traits made substantial progress in identifying genetic variants associated with these traits. Surprisingly, genes from many signaling and metabolic pathways whose roles in such traits were well established in experimental studies did not show statistically significant associations with these traits. This lack of consistency between results of genetic association studies of human data and information from experimental studies might be explained by the fact that biological mechanisms of complex traits regulation result from a complicated interplay between many genetic and non-genetic factors that may differ in humans and laboratory animals. Studying such interplay requires more sophisticated approaches than used in traditional genetic association studies that test the association of one SNP at a time with a given trait. It means that genetic association studies of multifactorial traits should include analysis of genetic interactions and collective actions of interactions of many genetic and non-genetic factors. To address this problem, many statistical approaches that aimed to detect associations of gene-gene (GxG) interactions have been proposed during the last decades.

Critical reviews of the methods, related software packages used to detect the interactions between genetic loci that contribute to human genetic diseases and the difficulties in determining the biological relevance of statistical interactions are provided in the papers^[27–30]^. Most recent reviews describe various extensions of the Multifactor Dimensionality Reduction (MDR) approach^[31–34]^, using entropy in genetic interaction analyses^[35,36]^, implementation of machine learning techniques to study epistasis^[28,37,38]^, as well as many other approaches that differ in definitions of genetic interaction, the accuracy of calculations, and in computation time^[39–45]^.

This paper used the INTERSNP software package^[46]^ that implements a logistic regression framework. This approach allows for evaluating associations of genetic interactions with complex traits in the presence of observed covariates. It has been successfully used in our earlier genetic analyses of genetic interactions^[47]^. Useful information about other methods can also be found in the review paper^[48]^.

Information about genes involved in multifactorial regulation of aging, health, and lifespan-related traits in laboratory animals serves as a source of useful insights concerning genetic mechanisms that might regulate these traits in humans. This information is used for selecting candidate medications appropriate for testing in clinical trials. The failure of many expensive clinical trials to identify a proper medication [e.g., in case of Alzheimer’s disease (AD)] indicates the need to find more reliable and less expensive ways of testing whether genetic connections detected in experimental studies exist in humans. It is proposed that such testing can be done by applying genetic epidemiological methods to available human data on genotyped individuals collected in human longitudinal and cross-sectional studies. The efficient analysis of such data requires a convenient conceptual framework that would allow the researchers to perform comprehensive analyses of biological mechanisms and effciently integrate research findings.

Stress-related conceptual framework allows for linking together stressors, sensors of stress signals, and genes from stress response pathways as key players in mechanisms of multifactorial regulation of complex traits.

The use of a stress-related conceptual framework might be beneficial for studying multifactorial regulation of complex traits because it allows for selecting and linking together non-genetic factors (e.g., associated with cellular stressors) and genes involved in cellular stress response (e.g., such as ISR). It has been recently shown that interplay between the *GCN2/EIF2AK4* gene that serves as one of the sensors of cellular stress signals in the ISR pathway and the gene *CHOP/DDIT3* involved in the regulation of autophagy and apoptosis may play a crucial role in the regulation of aging, health, and life span/survival traits in laboratory animals^[25,49]^. This observation allows us to hypothesize that interplay between these genes may influence these traits in humans. Testing this hypothesis using human data would be an important step forward in the translation of knowledge from experimental studies to humans. This is because the interplay between the *GCN2/EIF2AK4* and *CHOP/DDIT3* genes influences aging, health, and lifespan/survival traits in laboratory animals^[25,50,51]^ does not mean that these genes play the same roles in humans.

### Does the interplay between the *GCN2/EIF2AK4* and *CHOP/DDIT3* genes influence human lifespan?

This paper reviewed experimental evidence about genetic mechanisms that regulate the effects of cellular stress on aging, health, and lifespan/survival traits. These effects are manifested at the cellular level and involve genes from the ISR pathway. To illustrate our approach to the analysis of the effects of genetic interplay on these traits, two genes have been selected from the ISR pathway. One, the *GCN2/EIF2AK4* gene, becomes activated by several cellular stressors, including amino acid starvation (deprivation). The transformed signal sent from this gene (but not the initial stress signal) activates other genes in the ISR pathway, including the *CHOP/DDIT3* gene, which is the second gene selected for analysis. The product of this gene, among other things, influences the cells’ fate: when stress is mild, and the duration of the stress response is relatively short, the cell has high chances to survive; alternatively, under strong or persistent stress, the *CHOP/DDIT3* gene is more likely to activate the process of apoptosis for this cell. The strength and the duration of cellular stress response to a large extent might be determined by the *GCN2/EIF2AK4* gene polymorphisms. The cells’ fate has important consequences for the organism’s health and survival outcomes. Because of the importance of stress-related conceptual framework used in our analysis, a brief description of ISR is given below.

### Rationale for selecting the *GCN2/EIF2AK4* and *CHOP/DDIT3* genes involved in the ISR pathway

In response to various stressors disturbing normal cellular functioning, eukaryotic cells activate an evolutionary conserved adaptive machinery - the ISR^[49]^. Depending on the strength and duration of the stress response, ISR determines the fate of the cell^[52]^. Cellular stressors may be intrinsic (e.g., misfolded proteins, genetic polymorphisms^[53]^) or external (e.g., nutrient deprivation, viral infection, hypoxia, UV-irradiation, and others^[54–56]^). Experimental and clinical studies provide evidence about involvement of ISR in lifespan regulation^[57,58]^, as well as in the development of aging-related diseases including cognitive and neurodegenerative disorders^[49,55]^, cancer^[59–61]^, pulmonary disease^[62]^, atherosclerosis^[63,64]^, diabetes^[65]^ and other metabolic disorders^[66]^.

ISR responds to cellular stressors by changing the process of protein synthesis^[49,52]^. This process starts with the phosphorylation of eukaryotic translation initiation factor 2 alpha (EIF2A) by one of the four members of the EIF2A kinase family, which sense cellular stress signals. These include a heme-regulated inhibitor kinase (HRI/EIF2AK1), an interferon-induced, double-stranded RNA (dsRNA)-activated protein kinase (PKR/EIF2AK2), a protein kinase R (PKR)-like endoplasmic reticulum (ER) kinase (PERK/EIF2AK3), and a general control nonderepressible 2 kinase (GCN2/EIF2AK4). Additional details about these kinases are described below.

Heme regulating inhibitor kinase (HRI/EIF2AK1) is an enzyme that in humans is encoded by the *EIF2AK1* gene. Heme is an iron-containing compound that forms the non-protein part of hemoglobin, the substance inside red blood cells that binds to oxygen in the lungs and carries it to the tissues. HRI/EIF2AK1 is an intracellular heme sensor that coordinates heme and globin synthesis in erythropoiesis by inhibiting protein synthesis of globin and heme biosynthetic enzymes during heme deficiency. HRI is also activated by arsenite-induced oxidative stress, heat shock, nitric oxide, 26S proteasome inhibition, and osmotic stress. These types of stressors activate HRI independently of heme but require the presence of heat shock proteins HSP90 and HSP70. Denatured proteins and oxidative stress also activate HRI^[56,67,68]^.

Protein kinase R (PKR/EIF2AK2) is an enzyme that in humans is encoded by the *EIF2AK2* gene. In addition to dsRNA that can be introduced to the cell by a viral infection, PKR is also activated by oxidative and ER stress, growth factor deprivation, cytokines, bacterial infections, ribotoxic stress^[69]^, caspase activity in the early stages of apoptosis^[70]^. It can also be activated by the protein PACT (that in humans is encoded by the *PRKRA* gene) or by heparin and other cellular stress signals^[71–73]^.

Protein kinase R-like endoplasmic reticulum kinase (PERK/EF2AK3) is an enzyme that in humans is encoded by the *EIF2AK3* gene. PERK is activated by accumulation of misfolded (unfolded) proteins in the ER, perturbations in calcium homeostasis, cellular energy, mitochondrial stress (including uncoupling), or redox status^[74,75]^. It has also been reported to respond to ATP depletion and subsequent sarcoplasmic/ER Ca2+-ATPase pump inhibition in the context of glucose deprivation in neuronal cells and pancreatic β cells^[76–78]^. It initiates the unfolded protein response^[79,80]^. PERK plays an important role in Alzheimer’s and other neurodegenerative diseases^[81–83]^.

GCN2/EIF2AK4 is an enzyme that in humans is encoded by the *EIF2AK4* gene. GCN2 is evolutionarily conserved from yeasts to humans and plays a key role in modulating amino acid metabolism. It is activated in response to amino acid deprivation when it binds to deacylated transfer RNAs (tRNAs) via histidyl-tRNA synthetase-related domain^[25,84–87]^. GCN2 can also be activated by other stressors, including ultraviolet irradiation, viral infection, serum starvation, glucose deprivation, and oxidative stress. Recent work shows that GCN2 strongly activates by binding to ribosomal protein, suggesting that GCN2 actively monitors mRNA translation^[25,84–87]^. Recently, a pivotal role for GCN2 in response to membrane damage has been uncovered^[88,89]^. Finally, GCN2, a crucial regulator of amino acid metabolism, is necessary for the metabolic homeostasis of tumor cells. Tumors lacking GCN2 or ATF4 grow more slowly^[61]^. Thus, in cancers where amino acids are scarce, targeting the GCN2 branch of the ISR may be beneficial. Indeed, combination treatment with L-asparaginase and GCN2 inhibitors causes apoptosis in several cancer cell types^[49]^. GCN2 upregulates a coordinately expressed set of genes involved in amino acid biosynthesis and metabolism^[90]^.

The EIF2A phosphorylation by one of four kinases results in a decrease in global protein synthesis and the enhancing translation of the activating transcription factor ATF4 and several other genes acting together to restore cellular homeostasis^[91,92]^. ATF4 mediates the induction of ATF3 and GADD34/PPP1R15A, which dephosphorylates EIF2A-P and leads to the termination of ISR^[93,94]^. Chronic ISR activates CHOP/DDIT3 leading to apoptosis^[60,95]^. EIF2A phosphorylation also blocks the action of EIF2B, resulting in a general reduction in protein synthesis and the upregulation of selected genes. One of such genes is the transcription factor *ATF4*. The product of this gene plays a critical role in the regulation of obesity, glucose homeostasis, energy expenditure, and neural plasticity^[96,97]^.

Under stress conditions, increased ATF4 expression can activate several transcriptional programs that will ultimately determine the cell fate-from re-establishment of homeostasis to cell death^[98]^. The ability of ATF4 to interact with multiple other transcription factors makes its target genes highly dependent on stress intensity and cellular context^[61,87,99–101]^. For example, when acting in combination with ATF3, ATF4 contributes to re-establishing cellular homeostasis and survival promotion^[102]^. Conversely, when interacting with CHOP, ATF4 promotes cell death^[93]^. In addition to the interacting partners that cooperate with ATF4 to promote transcription of target genes, another set of interacting partners prevent ATF4 transcriptional activity, as is the case for PHD3 during hypoxia^[96,97]^ and TRIB3 during amino acid starvation^[103]^.

## METHODS

### Data

This paper aims to show how information from experimental data can be used for testing the connection between genetic factors and survival traits in humans. For this, a set of data is needed on genotyped individuals with large sample size. The Health and Retirement Study (HRS) data on white individuals satisfies this requirement. The information on white HRS study subjects is shown in Table 1.

The genetic data - 2.5 million single nucleotide polymorphisms (SNPs) - were produced on the Illumina platform using Illumina’s Human Omni2.5-Quad (Omni2.5) BeadChip methodology on 15,620 individuals (6472 males and 9148 females).

Quality control (QC) was performed before running the analysis using two procedures. The first, based on the protocol proposed in^[104]^, resulted in: 129 individuals dropped because of failure in the check of duplicates, missingness rate (5%), heterozygosity outlier (± 3 SD), sex mismatch, and divergent ancestry outlier (± 8 SD); 538,451 variants were removed due to minor allele frequency < 1%, 69,947 variants were removed due to genotyping missing rate higher than 5%; and 411,945 variants were removed based on Hardy-Weinberg test (HWE, *P*-value < 1.0E-7). The second procedure used the protocol proposed in^[105]^. Individuals and SNP which passed either the first QC step or the second step remained in the final dataset. This procedure allowed us to increase the number of study subjects used in genetic analysis. It led to 15,492 individuals and 1,267,439 variants cleaned and mapped to the human reference genome GRCh38 for further analysis.

The following dichotomous survival trait (ST) was used in genetic interaction analysis: *case*: LS ≥ 85; *control*: 75 ≤ LS < 85 (or age at last follow-up) because we were especially interested in the effects of genetic factors on survival at ages 85+ compared with that around age 80 (± 5 years). Our earlier studies^[106,107]^ suggested that the age around 80 might be a “switching point” in the course of aging, characterized by declining or leveling-off (after a prior increase) risks of some major diseases (e.g., cancers, asthma, CVD, diabetes). Such behavior of risk trajectories could be due to selection, under-diagnosis, or the aging itself, so that some aging-related changes in the body would negatively affect health and survival chances before age 80 but become protective afterward^[107]^. To estimate the association of interacting SNP pairs with ST, the logistic regression model as implemented in INTERSNP software was used (with the interaction term being the quantity of interest)^[46]^. Education, smoking status, sex, and first five principal components (PC1-PC5) were included as observed covariates.

### Evaluating collective association of interactive SNP pairs with the survival trait

Because of the multifactorial nature of age-related health and lifespan-related traits, the interactions of SNPs from the *GCN2/EIF2AK4* or *CHOP/DDIT3* genes with SNPs from many other genes may also contribute to these traits. The interacting SNP pairs associated with these traits can be detected in the genome-wide-like association studies of interacting SNP pairs in which one SNP from the *GCN2/EIF2AK4* or *CHOP/DDIT3* genes is fixed, and others are all SNPs available for a given dataset that passed the quality control procedure. The results of this analysis can be used for constructing a SNP-specific composite index that can measure the association of many interacting SNP pairs with health and lifespan-related traits. This measure extends the notion of polygenic risk score (PRS), widely used in the genetic epidemiological studies of age-related diseases and longevity^[108–116]^. A simple measure of such capacity for a given SNP* might be the number of detected associations of interacting SNP pairs (with a fixed SNP*) whose *P*-values did not exceed a given *P*-value threshold (similar to the genetic dose index described in ref^[113]^). This paper introduced a measure of interactive capacity of a given SNP* involved in the lifespan regulation called “SNP-specific Interaction Polygenic Risk Score” (SIPRS). The construction of such indices and some of their properties related to multifactorial regulation of AD are described in Yashin *et al.*^[47]^.

## RESULTS

### The number of pairs of interacting SNPs in the two genes

To evaluate the significance of associations of the SNPxSNP interactions with the survival trait, the number of tested SNP pairs is needed to know when deciding about the presence or absence of the association of the interacting SNP pair with the trait. This number depends on the population under study and the genotyping platform. In the HRS data, after the quality control procedure, the *GCN2/EIF2AK4* and the *CHOP/DDIT3* genes have 63 and 8 SNPs, respectively. The analysis of associations of these SNPs with the survival trait (ST) (defined in the *Data and methods* section) showed that the associations of each of the 71 SNPs with survival trait did not reach the nominal level (*P* ≤ 0.05) of statistical significance. This allows for the hypothesis that the contribution of these genes to survival might be realized through their interaction effects.

To evaluate associations of interaction between the *GCN2/EIF2AK4* and *CHOP/DDIT3* genes with survival traits, a set of 504 potentially interacting SNP pairs has been used to estimate the set of probabilities of the type I error, which would happen if the decision about the presence of association of each SNP pair with survival trait would be made. Note that this procedure did not involve the “decision making” about the presence or absence of association of the SNPxSNP interaction with the trait. Probabilities of type I error have been calculated for each SNP pair and, hence, no correction for multiple testing is needed at this stage. Note that the Bonferroni correction for multiple testing, in this case, would be 9.92E-05.

### Significant associations of interacting SNP pairs between the *GCN2/EIF2AK4* and *CHOP/DDIT3* genes with human survival trait

The presence of linkage disequilibrium (LD) between SNPs related to each of two genes allows for reducing the number of potentially interacting SNP pairs tested for their associations with lifespan and for increasing the Bonferroni correction threshold. For this, the LD regions in each gene and selected one SNP pair as a representative for each such region have been identified. In the analysis, the SNP pair representatives are those who have the smallest value of the type I error among SNP pairs in this region. This clumping procedure used *R*^2^ = 0.1 LD threshold and resulted in 8 independent SNP pairs: 8 independent SNPs from the *GCN2/EIF2AK4* gene and 1 independent SNP from the *CHOP/DDIT3* gene. It gives us a set of 8 SNP pairs that can now be used for testing the null hypothesis about the absence of association of the interacting SNP pairs with survival traits. The smallest *P*-value resulted from the analysis of the association of SNPxSNP interaction with survival trait is 3.80E-03 for SNP rs16970024 from the *GCN2/EIF2AK4* gene interacting with SNP rs697221 related to the *CHOP/DDIT3* gene [Table 2]. All other interactions have *P*-values exceeding 1.00E-03. With eight SNP pairs tested in this analysis, the Bonferroni correction provides us with the *P*-value threshold 6.25E-03 for testing the null hypothesis, which is larger than 3.80E-03. These results are summarized in Table 2.

Separate analysis of males and females revealed a significant association of interactions between the SNPs rs16970024 and rs697221 with female survival, with a *P*-value of 3.80E-03. No significant associations of the interacting SNPs from these two genes with survival traits were detected in males.

These results support our hypothesis induced by the results of experimental studies that interactions between *GCN2/EIF2AK4* and *CHOP/DDIT3* genes may contribute to survival in humans.

### SNP-specific interaction polygenic risk scores for the rs16970024 and rs697221 SNPs

It is important to note that regulation of aging, health, and lifespan-related (survival) traits in humans may include genes and connections which are not detected in experimental studies with laboratory animals. Therefore testing associations of interactions between each of two detected SNPs and all other SNPs using available human data might result in the detection of new features of genetic human mechanism of lifespan regulation. It is because each of the detected SNPs may influence lifespan through its interactions with many other SNPs.

The number of significant associations with other SNPs and the strength of such associations may differ for each SNP. Measuring the “interacting ability” of a SNP might help better understand the contribution of a given SNP (and corresponding gene) to multifactorial regulation of lifespan. The convenient measure of such ability could be SNP-specific interaction polygenic risk score (SIPRS)^[47]^

Such measures have been calculated for each of the two detected SNPs. For this, genome-wide association study (GWAS)-like analysis of associations of the rs16970024 and then rs697221 SNPs with all other SNPs available in the HRS data were performed. The HRS data on white males and females combined was used in the analysis. The logistic regression model with the interaction term has been used for evaluating associations of interacting pairs of SNPs with survival traits. Education, smoking, sex, first five principal components PC1-PC5, and rs16970024 (rs697221) were used as observed covariates in the regression model. Then by using summary statistics resulted from these analyses, the rs16970024 (the rs697221) composite SIPRSs indices have been constructed using the procedure described in^[47]^.

**Figure 1.**
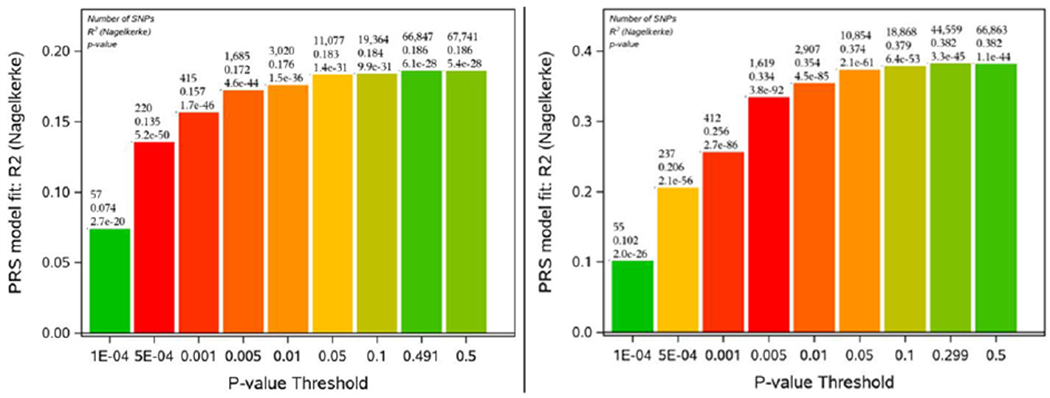
The diagrams illustrating properties of composite indices SIPRS constructed for the rs16970024 SNP (on the left) and SIPRS for rs697221 SNP on the right. The vertical axis represents proportions of phenotypic variances (*R*^2^) explained by the composite indices. Each pillar characterizes a version of the SIPRS index corresponding to the *P*-value threshold (shown under the pillar at the horizontal axis). Nine pillars at each diagram correspond to nine SIPRSs indices that summarize associations of different numbers of SNP pairs with survival traits. The numbers on the top of each pillar describe properties of the corresponding index: (1) the first line on the top shows the number of SNP pairs corresponding to the *P*-value threshold (shown below this pillar at the horizontal axis); (2) the second line from the top shows the value of *R*^2^; (3) the third line from the top represents the *P*-value of association of this SIPRS index with survival trait.

The construction of rs16970024 (rs697221) related indices included a set of 67,741 (66,863) interacting pairs of SNPs ranked with respect to *P*-values of their associations with survival trait (from smallest to the largest). Both positive and negative associations with survival traits were used in the construction of SIPRS indices. Then the properties of constructed SIPRS indices were investigated. The results of these analyses are shown in Figure 1.

The diagram on the left of Figure 1 shows that the rs16970024 SIPRS index corresponding to the threshold 5E-04 (horizontal axis under the pillar) has the smallest *P*-value of its association with survival trait *P* = 5.2E-50. This index contains 220 SNP pairs and explains 13.5% of phenotypic variance of the survival trait. The second most significant rs16970024 related index shown in this diagram corresponds to the threshold 0.001 with the *P*-value 1.7E-46 of its association with survival trait. This index contains 415 SNP pairs and explains 15.7% of phenotypic variance of the survival trait.

The diagram on the right of this figure shows that the rs697221 SIPRS index corresponding to the threshold 0.005 (horizontal axis under the pillar) has the smallest *P*-value of its association with survival trait *P* = 3.8E-92. This index contains 1619 SNP pairs and explains 33.4% of phenotypic variance of survival traits. The second most significant rs697221 SIPRS index corresponds to the threshold 0.001 (at horizontal axis under the pillar) with the *P*-value 2.7E-86 of its association with survival trait. This index contains 412 SNP pairs and explains 25.6% of phenotypic variance of the survival trait.

The R package pROC was used to calculate the areas under the receiver operating characteristics curves (AUC) for selected rs16970024 (rs697221) SIPRS indices with a corresponding threshold of 0.001. The AUC characterizes the fit of the logistic regression model describing the association of the rs16970024 (rs697221) specific SIPRS constructed from 415 (412) SNP pairs (the threshold value 0.001). The area under the curve (AUC) is 0.64 with 95% of confidence interval (0.63-0.66) for rs16970024 SIPRS, and 0.71 with 95% of confidence interval (0.70-0.72) for rs697221 SIPRS, respectively.

The properties of interacting SNP pairs most significantly associated with survival trait in which the rs16970024 SNP interacts with other SNPs are presented in Supplementary Table 1. The properties of interacting SNP pairs most significantly associated with survival trait in which the rs697221 SNP interacts with other SNPs are presented in Supplementary Table 2.

## DISCUSSION

The hypothesis-free GWAS of SNPxSNP interactions, including SNPs from all selected candidate genes, is possible but involves testing many SNPxSNP interactions. The too-conservative Bonferroni correction for multiple testing often results in unjustified decisions like “we consider all associations having *P*-value smaller than 5.0E-03, (5.0E-04, 5.0E-05) as promising” may make the interpretation of the results of such analysis difficult. At the same time, useful insights about the potential role of interplay between specific pairs of genes in the trait of interest might be obtained from experimental studies. Testing the presence of such connection in humans can be done by estimating the association of SNPxSNP interactions with the trait using SNPs taken only from given two genes. It is important to note that, even in this case, the number of testing SNP pairs can be large enough to create problems with deciding on true-positive association. It turns out that the number of testing SNP pairs can be further reduced using the fact that many SNPs from these two genes are in LD. Making such steps increases the chances of finding true-positive associations without making assumptions which compromise statistical evidence. This paper showed how such analysis could be done. Two candidate genes have been selected, which play important roles in the ISR pathway. One, the *GCN2/EIF2AK4* gene, is a sensor of amino acid starvation. Other, the *CHOP/DDIT3* gene is involved in the regulation of apoptosis and autophagy. The hypothesis has been tested that the interplay between these genes may contribute to variability in human lifespan. Using data on HRS study participants, it has been found that interaction between SNP rs16970024 from the *GCN2/EIF2AK4* gene and SNP rs697221 from the *CHOP/DDIT3* gene is significantly associated with human survival traits for females and for males and females combined.

### Geroscience and the ISR pathway

The geroscience hypothesis is that slowing down the aging process will postpone the occurrence of many age-associated health disorders, which results in increased healthspan and improved survival^[117]^. The idea to understand and control the individual aging process motivated many researchers to study biological mechanisms of aging and search for possible interventions that could slow down this process. One class of the interventions affecting aging has been discovered in the first half of the last century by McCay *et al*.^[118]^ in experiments studying the effects of dietary restriction on lifespan in rodents. Subsequent experiments showed that a CR/DR diet is able to improve health and increase lifespan in different animal models^[119,120]^.

More recent studies identified genetic pathways that sense disturbances in nutrients supply, regulate metabolic functions in CR/DR conditions, and influence aging, health, and lifespan^[121]^. A part of such regulation in laboratory animals is realized through the ISR pathway, in which interplay between the *GCN2/EIF2AK4* and *CHOP/DDIT3* genes dealing with the response to nutritional stress may play an essential role. Nutritional stress was an important part of life in human ancestors. This explains a major role of the sensor of the amino acid starvation (the *GCN2/EIF2AK4* gene) in the ISR regulation of the processes affecting aging, health, and lifespan-related traits in response to changes in nutritional status. Experimental studies demonstrated a high potential of genes from the ISR pathway as targets for pharmacological intervention^[122–128]^. These studies indicated that evaluating the role of ISR pathway in human aging and lifespan may substantially improve our understanding of the factors and mechanisms initiating the development of major human age-associated health disorders.

### Amino acid starvation

It was found that several mechanisms involved in sensing and regulation of response to amino acid (AA) deprivation can improve health and increase lifespan^[129]^. The *GCN2/EIF2AK4* and *mTORC1* genes are both involved in such regulation. The key role of mTOR complex 1 (mTORC1) signaling pathway regulation of aging and lifespan has been established and widely discussed in the literature^[23,26,130–133]^. Decreased activation of mTORC1 leads to lifespan extension in yeast, worms, flies, and mice^[134]^.

It turns out that the mechanism is driven by the AA deficiency sensor GCN2/EIF2AK4 also influences aging, health, and lifespan/survival traits ^[25,135–137]^. The activation of the ISR in response to nutrient starvation engages adaptive changes mediated by the induction of genes necessary to produce all the amino acids^[138]^. Amino acids are needed to maintain various cellular functions, including the Krebs cycle activity for ATP generation. They also provide necessary components for maintaining redox homeostasis^[138]^. These properties of AA regulation can be used to deal with the consequences of metabolic stress.

### Insights from experimental studies should be tested using human data

The majority of information about how the interplay of genes from the ISR pathway may influence aging, health, and lifespan/survival traits is obtained in experimental studies of these traits. Even though many genetic stress response pathways are evolutionary conserved, the biological processes that involve groups of such genes in humans may differ from those developing in laboratory animals or cellular cultures. This is because, in different species, such pathways may experience species-specific modifications, acquire some, and lose other functions when adjusting to a specific biological background, nutritional differences, and external conditions. Therefore, the fact that an interplay between the *GCN2/EIF2AK4* and *CHOP/DDI3T* genes (the members of the ISR pathway) influences lifespan/survival of laboratory animals does not mean that the same connection holds in humans. Genetic epidemiological analysis of available data on genotyped human individuals has been performed to test whether interplay between these genes is associated with human lifespan/survival. It has been found that one interacting SNP pairs taken from the *GCN2/EIF2AK4* and *CHOP/DDI3T* genes showed a statistically significant association with human survival trait in the analyses of the HRS population of males and females combined. Sex-specific analysis revealed that a statistically significant association of interacting SNPs with survival is confirmed only in females. This result may indicate that survival in males and females are regulated using different biological mechanisms. It can also result from the fact that the population of males used in the analysis was smaller than females. This finding is an important step in the process of translation of the results of experimental studies to human applications.

### Detected association of the interaction between two genes with the human survival motivates search for biological mediators of such connection

The association of the interaction between GCN2/EIF2AK4 and CHOP/DDIT3 with the human survival trait detected in statistical analysis of data does not necessarily mean that this trait is affected by the result of biochemical interaction between corresponding genetic products. Statistical analysis may capture genetic connections between two genes that could be mediated by a chain of biochemical reactions that involve products of many other genes from ISR and other signaling and metabolic pathways involved in regulating a given survival trait in humans. Identifying such mediators and evaluating their roles in the regulation of human aging, health, and survival traits could shed light on the mechanism of multifactorial regulation of these traits in humans.

The ISR pathway that activates the *GCN2/EIF2AK4* and *CHOP/DDIT3* along with other genes may influence the development of age-associated diseases, including cancer^[59,139,140]^, neurodegeneration^[47]^, diabetes^[141]^, other^[25]^, and through them, lifespan and survival traits. Experimental data also show that the *GCN2/EIF2AK4* and *CHOP/DDIT3* genes can be involved in the regulation of autophagy and apoptosis^[142–146]^, which play a fundamental role in cancer^[147,148]^, bacterial infections^[149]^, other health disorders^[150–156]^, aging^[157–159]^. The trade-off regulation between autophagy and apoptosis at the cellular level might be responsible for variability in lifespan[143,160]. Genes involved in such regulation are potential mediators of statistically detected association of interaction between the *GCN2/EIF2AK4* and *CHOP/DDIT3* genes and the survival trait.

### ISR initiation may improve or deteriorate health and survival outcomes

Experimental studies provide evidence about both the positive and negative influence of the ISR initiation on health and survival traits[57,58,62]. The improvement in survival is likely to be related to the reduction of the metabolic rate in the cells at the time of cellular stress response, which is in concert with the Max Rubner’s “rate of living theory of aging”^[161]^. Experiments confirming the positive effects of CR/DR on aging, health, and lifespan illustrate this property^[162–169]^. The mechanism responsible for the positive effect on survival might be related to the fact that both genes analyzed in this paper are involved in the regulation of autophagy^[142,152]^. The negative correlation between basal (resting) metabolic rate and human lifespan was also detected^[170,171]^. All these effects were likely to be manifested, because in most of cells exposed to stress the ISR ended up by restoration of normal cellular functioning. The deleterious effects of the cellular stress response on these traits are likely to be manifested when ISR contributes to the survival of malignant cells or produces destruction of post-mitotic cells by apoptosis.

### Earlier studies of the association of genetic interaction with human survival and longevity

The association of GxG interaction with human longevity has been investigated in several earlier studies. Tan *et al*.^[172]^ suggested a centenarian-only approach for assessing such connections. Using data from Italian centenarians, the authors detected an association of interaction between the *REN* gene and the mitochondrial H haplotype with longevity. The case-control study of Han Chinese centenarians found that the interactions of SNPs from the *FOXO1A* and *FOXO3A* genes are associated with survival. This study also found that the interaction of FOXO1A and regular exercise is associated with survival traits^[173]^. The role of interaction between SNPs from the *FOXO1A* and *FOXO3A* genes with longevity has also been studied by implementing a novel permutation test to the data from the Danish 1905 birth cohort^[174]^. The analysis confirmed the association of interaction of SNPs from these genes with longevity; however, interacting SNPs detected in this study differed from those found in the study of Chinese centenarians^[173]^. Dato *et al.*^[175]^ analyzed associations of interacting SNPs from candidate genes on longevity using data from the Danish 1905 cohort. Curk *et al.*^[176]^ used an information-theoretic approach implemented in the SNPsyn software and the MDR method to select synergistic pairs of SNPs. The best combinations detected in both approaches included SNPs from *IGFR* and *PTPN1, TP53*, and *ERCC2, TXNRD1* and *TP53.* The authors also found interacting partners: *PAPPA, PTPN1, MRE11A*, and *PARK7* for the *GHSR* gene previously identified in a single-SNP association study. Ukraintseva *et al.* (forthcoming) investigated associations of interactions of SNPs from the group of candidate genes from aging-related pathways (IGF/FOXO growth signaling, P53/P16 apoptosis/senescence, and MTOR/SK6 mediated autophagy) with survival traits. For this, the INTERSNIP software for epistasis analysis was used in the analysis of data from the Atherosclerosis Risk in Communities study. The results of this analysis suggest that the interactions between SNPs from the *IGF1R* and *TGFBR2* genes, as well as SNPs from the *BLC2* gene, may influence human lifespan. These results were validated using data from the Cardiovascular Health Study. The results of these analyses showed that interactions between SNPs in genes from aging pathways have higher associations with survival traits than individual SNPs for the same genes^[177]^.

### Why our analysis has higher chances of rejecting the null hypothesis about the absence of true-positive associations of SNPxSNP interaction with survival trait

The hypothesis-free genome-wide association study (GWAS) of SNPxSNP interactions, including SNPs from all selected candidate genes from the ISR pathway, is possible but involves testing of many SNPxSNP interactions. The too-small *P*-value threshold for making the decision about the presence of true-positive association resulting from the Bonferroni correction for multiple testing often becomes responsible for statistically unjustified decisions like “we consider all associations having *P*-value smaller than 5.0E-03, (5.0E-04, or 5.0E-05) as promising” may make the interpretation of the results of such analysis doubtful. At the same time, useful insights about the potential role of interplay between specific pairs of genes in the trait of interest might be obtained from the results of experimental studies. Testing the presence of such connection in humans can be done by estimating the association of SNPxSNP interactions with the trait using SNPs taken only from selected two genes. It is important to note that even in this case, the number of testing SNP pairs can be large enough to create problems with deciding on true-positive association. It turns out that the number of testing SNP pairs can be further reduced using the fact that many SNPs from these two genes are in LD. Making such steps increases the chances of rejecting the null hypothesis about the absence of associations without making assumptions that compromise statistical evidence.

### The use of LD for the reduction of the number of comparing SNP pairs

This paper showed how such analysis could be done. Two candidate genes have been selected, which play important roles in the ISR pathway. One, the *GCN2/EIF2AK4* gene, is a sensor of amino acid starvation. Other, the *CHOP/DDIT3* gene is involved in the regulation of apoptosis and autophagy. All SNPs in each gene have been divided into subsets of SNPs that are in LD with each other and whose LD measure *R*^2^ was larger or equal to a given threshold (in our case, *R*^2^ ≥ 0.1). This procedure divided all available SNP pairs into corresponding subsets. Note that the smaller is the LD threshold, the larger number of correlated SNPs that could be included in the subsets, and the smaller number of such subsets will be constructed. One representative SNP pair was selected for each subset of SNPs in LD to reduce the number of comparing SNP pairs. In our analysis, the SNP pair representatives were those who have the smallest value of the type I error among SNP pairs in this subset. This procedure resulted in 8 independent SNP pairs constructed from 8 independent SNPs from the *GCN2/EIF2AK4* gene and 1 independent SNP from the *CHOP/DDIT3* gene. Then this set of 8 SNP pairs was used for testing the null-hypothesis about the absence of association of the interacting SNP pairs with survival trait. Using data on HRS study participants on males and females combined, and then separately for males and females, it has been found that the null-hypothesis about the absence of association of the interaction between SNP rs16970024 from the *GCN2/EIF2AK4* gene and SNP rs697221 from the *CHOP/DDIT3* gene with survival trait can be rejected for females and for males and females combined, which means that the interaction of these SNPs is significantly associated with human survival trait.

### The SNP specific interaction polygenic risk scores

The mechanism of multifactorial regulation of human lifespan is largely unknown. Therefore, each SNP from detected SNP pair may interact with SNPs from many other genes outside ISR pathway, and these interactions may also contribute to lifespan and survival traits. GWAS-like procedure has been used in which one of the detected SNPs is fixed, and others include all available SNPs that passed the quality control procedure. The results of this analysis were used for constructing a SNP-specific measure of collective association of many interacting SNP pairs, called the SIPRS, with survival trait. Using such an index, one gets an opportunity to evaluate collective associations with the trait of interest of different numbers of interacting SNP pairs in which a given SNP interacts with other SNPs. The approach allows for the construction of many ordered indices for a given SNP that consist of different numbers of interacting SNP pairs [Figure 1].

The properties of these indices can be compared, and the most appropriate can be selected for further analysis. It has been found that the rs16970024 SIPRS index constructed from 220 most significant interacting SNP pairs has the most significant association with survival trait with *P* = 5.2E-50. Index constructed from 57 most significant interacting SNP pairs is less significant with *P* = 2.7E-20 but contains a smaller number of SNP pairs and, hence, might be more convenient for starting a further analysis.

It has also been found that the rs697221 SIPRS constructed from 1619 most significant SNP pairs has the most significant association with survival trait with *P* = 3.8E-92. Index constructed from 55 SNP pairs is less significant with *P* = 2.E-26 [Figure 1] but contains a smaller number of SNP pairs and, hence, might be more convenient for further analysis.

Supplementary Table 1 describes properties of interacting SNP pairs in which interactions of the rs16970024 SNP with other SNPs were associated with survival trait with *P*-value not exceeding 9.69E-05. These interactions were used in the construction of the SIPRS for the rs16970024 SNP. Supplementary Table 2 describes properties of interacting SNP pairs in which interactions of the rs697221 SNP with other SNPs were associated with survival trait with *P*-value not exceeding 9.97E-05. These interactions were used in the construction of the SIPRS for the rs697221 SNP.

## LIMITATIONS

### Multifactorial regulation of complex traits involves interplay among many genes

Results of this study suggest that the interplay between the two key genes from the ISR pathway involved in aging, health, and lifespan traits in laboratory animals can also be involved in the regulation of human survival. This observation does not explain the entire mechanism of the multifactorial regulation of human lifespan, which can involve many other interacting genes. However, the use of only two genes of the many, allowed us to reduce the number of statistical tests and provide a proof of concept that the connection between genes and lifespan discovered in laboratory animals can also take place in humans, in the form of genetic interactions. Experimental aging studies found many other genes whose interplay may also contribute to aging, health, and survival traits. However, detecting the effects of multiple interacting genes on lifespan remains a challenging problem. Its solution requires very large sample sizes of study participants and/or advanced methods of analysis. The latter can be partly addressed by constructing and investigating the SIPRSs^[47]^.

### Dynamic regulation of aging, health, and survival traits

Individual lifespan and health status are outcomes of dynamic processes in which the interacting genetic and non-genetic factors, the strength of their interactions, and other influential variables can change with age, time, and other conditions. Genetic interaction analyses that have been performed so far, including in this study, are initial steps in addressing the multifactorial nature of complex traits and do not yet include the dynamic properties of these traits in the analysis. More efficient methods of a comprehensive statistical analysis of the dynamic polygenic regulation of the aging, health, and survival traits are urgently needed to improve our understanding of these traits substantially. Merging biodemographic methods of statistical modeling with genetic epidemiology and systems biology of aging may be a promising way to address this problem. These methods can be developed within the stress-related conceptual framework linking components of individual resistance to stresses, including robustness (vulnerability) and resilience (ability to recover)^[178–182]^, allostatic adaptation and allostatic load^[183,184]^ with health and survival outcomes.

### From association to causality

The associations of interacting SNP pairs with survival traits evaluated in this paper do not necessarily describe causal connections. This limitation is common for statistical methods used in genetic analyses of observational data. Information about causal connections is needed for developing intervention therapies, testing candidate medications. Testing causality of connections detected in the analysis of observational data can be performed using methods of mediation analysis^[185]^ and Mendelian randomization^[186]^. These methods have their own limitations^[187]^.

### Lack of information about cellular stressors in human data

The use of the stress-related conceptual framework in studies of multifactorial mechanisms of lifespan regulation would benefit from information about biomarkers characterizing cellular stressors, as well as about factors and conditions capable of affecting variability (e.g., strength, duration) of the cellular stress response. Since many human data on aging, health, and lifespan related traits have limited information about such stressors, factors, and conditions, some useful insights and ideas about possible stressors can be obtained indirectly from the estimates of the roles of specific genetic sensors of cellular stress signals in lifespan regulation obtained in data analyses. This is because each such sensor recognizes and responds to specific (sometimes overlapping) groups of stress signals.

### Genetic interactions: statistical vs. biological epistasis

Genetic interactions may produce paradoxical results in the genetic association analyses of complex traits^[107,188–191]^. One should, however, distinguish between biological and statistical epistasis. In biological experiments, epistasis may be detected as a result of proteins’ interactions, in which the effect of one protein can mask the effect of another protein on the phenotype of interest^[107]^. In genetic epidemiology, the effects of genetic interactions on the trait of interest are evaluated using regression models with the interaction terms^[46]^. Since these genetic epidemiology methods differ from those used in detecting the effects of epistasis in biological experiments, the results of such analyses may also have different interpretations^[107,192]^. Associations of genetic interactions with survival traits detected in epidemiological studies may reflect synergistic or antagonistic effects of genes whose products do not directly influence each other and, therefore, will not necessarily be detected in studies of biological epistasis. In contrast to biological epistasis, statistical interactions can capture connections between genes mediated by the chain of intermediate genetic products. Identification of such intermediate genes and evaluation of their roles in aging, health, and lifespan may require new experimental studies. The detected statistical association does not exclude the presence of the effect of biological epistasis between two genetic products on human lifespan. However, the association of statistical genetic interaction with survival may be detected even in the absence of direct biological interaction between genetic products. This might be the case when such interaction and connection with the trait are mediated by other genes.

### Long road to understanding multifactorial mechanism regulating human lifespan

The results of this paper do not provide us with all the details about mechanisms of multifactorial regulation of human lifespan. They, rather, describe a way to become more informative about it. An important question that was not addressed in this study is how the confirmed (or newly detected) information about multifactorial regulation of aging, health, and survival in humans should be integrated to explain health and survival outcomes? To be seful in practice, mechanisms of such integration should have quantitative description, e.g., in the form of a computer model capable of describing the response of the body (cells, organs, systems) to specific challenges. A promising tool for addressing this problem could be further development of dynamic stochastic modeling of human mortality and aging, which successfully used in the analysis of longitudinal data^[182]^.

### Conclusion

Experimental studies pointed to a fundamental role of the interplay between the *GCN2/EIF2AK4* (involved in sensing cellular stress signals) and *CHOP/DDIT3* (involved in apoptosis and autophagy), as well as other genes from the ISR pathway, in aging, health, and lifespan of laboratory animals^[25,193]^, which encourages clarifying the role of interactions among these genes in respective human traits.

Results of this study support our hypothesis that the interplay between *GCN2/EIF2AK4* and *CHOP/DDIT3* genes involved in the ISR pathway may influence lifespan in humans. This is a “proof of concept” research and a step forward to translating the evidence about mechanisms of lifespan regulation found in laboratory animals to humans.

Individual differences in the exposure to stressful conditions, in the access to health care, the polymorphisms in genes that sense cellular stress signals, and in other genes involved in cellular stress response, together with aging-related changes in robustness and resilience, are likely to be important sources of disparity and heterogeneity in health, lifespan and survival outcomes. Being evaluated, these characteristics can be used in predicting the future burden of aging-related diseases under different scenarios/strategies of reducing disparities, improving access to health care facilities and medical advances for the groups of individuals having different genetic backgrounds and exposed to distinct environmental and living conditions.

## Supplementary Material

Supplementary Table 1 and Table 2

## Figures and Tables

**Table 1.** Summary statistics for white HRS respondents with genetic data

	Males		Females		Total	

	Case	Control	Case	Control	Case	Control
***N***	1056	1592	1508	1986	2564	3578
**Dichotomous variables: *N* (Percentage)**						
Education, Highschool+	440 (91.06)	536 (94.07)	803 (94.29)	818 (96.91)	1243 (92.95)	1354 (95.63)
Ever smoked	703 (67.60)	845 (70.53)	615 (41.84)	759 (52.13)	1318(52.51)	1604 (60.44)

**Table 2.** Associations of interactions between SNPs in *GCN2/EIF2AK4* and *CHOP/DDIT3* genes with *survival trait after clumping with threshold *R*^2^ = 0.1

rsid1	1EA/NEA	1MA	p1	1MAF	rsid2	2EA/NEA	2MA	p2	2MAF	b12	p12
**rs16970024**	**A/G**	**G**	**0.81**	**0.06**	**rs697221**	**G/A**	**A**	**0.98**	**0.14**	**0.60**	**3.88E-03**
rs72731410	G/A	A	0.97	0.03	rs697221	G/A	A	0.98	0.14	0.75	2.81E-02
rs3736290	A/C	C	0.08	0.47	rs697221	G/A	A	0.98	0.14	0.19	5.20E-02
rs7169266	A/G	G	0,60	0.03	rs697221	G/A	A	0.98	0.14	−0.40	1.13E-01
rs76182620	A/G	G	0.54	0.05	rs697221	G/A	A	0.98	0.14	−0.34	1.35E-01
rs12442731	A/G	G	0.17	0.44	rs697221	G/A	A	0.98	0.14	−0.14	1.87E-01
rs117584784	G/A	A	0.44	0.02	rs697221	G/A	A	0.98	0.14	−0.21	4.82E-01
rs566792	G/A	A	069	0.13	rs697221	G/A	A	0.98	0.14	−0.05	0.731

Notations for the columns: rsid1 and rsid2 denote the SNP names from the *GCN2/EIF2A4* and *CHOP/DDIT3* genes, respectively; 1EA/NEA, 2EA/NEA; 1MA, 2MA; p1, p2 denote the effect/non-effect alleles; minor alleles for SNPs; and *P*-values for individual associations of SNPs from columns rsid1 and rsid2, respectively. Terms b12 and p12 denote the regression coefficients and *P*-values of associations between interacting SNP pairs and survival traits, respectively.

* Survival trait: LS ≥ 85 (“case”); and 75 ≤ LS or age at the last follow-up < 85 (“control”).

Covariates: education, smoking status, sex, first five principal components. MAF: minor allele frequency.

## Data Availability

Not applicable.
