# Supplementary material for: Roles of interacting stress-related genes in lifespan regulation: insights for translating experimental findings to humans
Source: J Transl Genet Genom. Author manuscript; Available in PMC 2021 Nov 24. (PMC8612394)
Supplement: Supplementary Table 1 and Table 2 [file NIHMS1749829-supplement-Supplementary_Table_1_and_Table_2.docx]

**Supplementary Materials**

**Roles of interacting stress related genes in lifespan regulation:**

**Insights for translating experimental findings to humans**

Anatoliy I Yashin^1^, Deqing Wu^1^, Konstantin Arbeev^1^, Arseniy P Yashkin^1^, Igor Akushevich^1^, Olivia Bagley^1^, Matt Duan^1^, Svetlana Ukraintseva^1^

*^1^Biodemography of Aging Research Unit, Duke University, Durham, NC 27705, USA*

**Table S1.** SNPs whose interaction with rs16970024 SNP were associated with survival trait with p-value not exceeding 9.69E-05. Notations: “rsid” is SNP name; “chr” denotes chromosome number; “EA” is an effective allele; “AA” is an alternative allele; “MA” denotes minor allele; “pS” denotes p-value in separate genetic analysis of association of a given SNP with survival trait; “MAF” is a minor allele frequency; gene is used for gene name; “b12” is a beta coefficient characterizing association of the interaction of the rs697221 SNP with a SNP in the “rsid” column with survival trait; “p12” is the p-value of association between interaction of the rs16970024 SNP with a SNP shown in the “rsid” column.

| rsid | chr | EA | AA | MA | pS | MAF | gene | b12 | p12 |
| --- | --- | --- | --- | --- | --- | --- | --- | --- | --- |
| rs13023094 | 2 | A | C | C | 0,807 | 0,217 | SLC4A1AP | 0,6458 | 3,39E-06 |
| rs4388939 | 12 | A | G | G | 0,0223 | 0,067 | FICD | -0,9674 | 8,27E-06 |
| rs67998788 | 2 | A | G | G | 0,13 | 0,23 | 2q37.3 | 0,5781 | 8,78E-06 |
| rs17447148 | 1 | G | A | A | 0,403 | 0,101 | 1p31.1 | 0,8952 | 9,97E-06 |
| rs1409813 | 1 | G | A | A | 0,0214 | 0,301 | TSEN15 | -0,5404 | 1,25E-05 |
| rs2035892 | 8 | G | A | A | 0,842 | 0,103 | C8orf37-AS1 | 0,8204 | 1,60E-05 |
| rs7550206 | 1 | G | A | A | 0,141 | 0,24 | 1p22.2 | -0,5554 | 1,67E-05 |
| rs68176631 | 9 | A | G | G | 0,909 | 0,349 | LINGO2 | -0,5089 | 1,67E-05 |
| rs2433644 | 12 | C | A | A | 0,981 | 0,385 | 12p12.1 | -0,4841 | 1,85E-05 |
| rs1876801 | 2 | A | G | G | 0,0967 | 0,134 | LOC105373514 | 0,7545 | 1,86E-05 |
| rs79742301 | 21 | G | A | A | 0,771 | 0,138 | 21q22.11 | -0,6653 | 2,52E-05 |
| rs10880841 | 12 | C | A | A | 0,0304 | 0,208 | 12q12 | -0,5531 | 2,61E-05 |
| rs1317639 | 3 | A | G | G | 0,417 | 0,255 | PTPRG | -0,5251 | 2,71E-05 |
| rs12434716 | 14 | C | G | G | 0,299 | 0,164 | NPAS3 | 0,6714 | 2,82E-05 |
| rs77323270 | 15 | G | A | A | 0,184 | 0,053 | ANPEP | 1,0528 | 2,91E-05 |
| rs7569776 | 2 | G | A | A | 0,381 | 0,431 | 2q21.1 | 0,4532 | 3,01E-05 |
| rs840716 | 11 | C | A | A | 0,973 | 0,053 | OR51G1 | 1,0883 | 3,08E-05 |
| rs4245284 | 18 | A | G | G | 0,885 | 0,222 | 18q21.32 | 0,5612 | 3,08E-05 |
| rs10512751 | 5 | A | C | C | 0,157 | 0,165 | C7 | -0,5881 | 3,24E-05 |
| rs11973636 | 7 | G | A | A | 0,61 | 0,392 | LOC107986820 | -0,4458 | 3,25E-05 |
| rs12932507 | 16 | T | A | A | 0,68 | 0,104 | CDH13 | -0,7429 | 3,32E-05 |
| rs62080360 | 17 | G | A | A | 0,479 | 0,114 | PGS1 | -0,6904 | 3,41E-05 |
| rs7238337 | 18 | G | A | A | 0,0697 | 0,283 | LOC107985165 | -0,5043 | 3,56E-05 |
| rs1475149 | 6 | G | A | A | 0,194 | 0,44 | CASC15 | -0,4576 | 3,60E-05 |
| rs7662014 | 4 | G | A | A | 0,929 | 0,104 | 4p14 | -0,756 | 3,80E-05 |
| rs13042739 | 20 | A | G | G | 0,258 | 0,124 | LOC112268271 | 0,7209 | 4,13E-05 |
| rs113061187 | 15 | A | G | G | 0,684 | 0,054 | SLCO3A1 | 1,0064 | 4,29E-05 |
| rs1451758 | 16 | G | A | A | 0,341 | 0,143 | LINC02165 | 0,6378 | 4,58E-05 |
| rs6975186 | 7 | A | T | T | 0,489 | 0,292 | CNTNAP2 | -0,4902 | 5,25E-05 |
| rs6507199 | 18 | A | G | G | 0,403 | 0,253 | CELF4 | 0,5202 | 5,40E-05 |
| rs731671 | 20 | G | A | A | 0,468 | 0,402 | PPP1R16B | 0,4386 | 5,56E-05 |
| rs2175094 | 2 | G | A | A | 0,207 | 0,218 | LOC105374382 | 0,5693 | 5,65E-05 |
| rs60730012 | 5 | G | A | A | 0,808 | 0,117 | LOC105374737 | -0,6488 | 5,99E-05 |
| rs2929965 | 8 | G | A | A | 0,0223 | 0,427 | CCN4 | 0,4742 | 6,38E-05 |
| rs6790160 | 3 | A | G | G | 0,721 | 0,086 | 3q13.13 | -0,773 | 6,39E-05 |
| rs11067978 | 12 | A | G | G | 0,85 | 0,144 | 12q24.21 | 0,6504 | 6,43E-05 |
| rs6557452 | 6 | G | A | A | 0,978 | 0,08 | LOC101928923 | 0,8895 | 6,65E-05 |
| rs10838329 | 11 | G | A | A | 0,957 | 0,264 | LINC02704 | 0,5049 | 6,75E-05 |
| rs11191157 | 10 | G | A | A | 0,455 | 0,287 | ARMH3 | -0,4897 | 6,99E-05 |
| rs1938548 | 6 | G | A | A | 0,392 | 0,11 | 6q14.1 | -0,6792 | 6,99E-05 |
| rs13153696 | 5 | C | A | A | 0,571 | 0,178 | CARMN | 0,5903 | 7,04E-05 |
| rs1118290 | 9 | G | A | A | 0,898 | 0,349 | LOC105375951 | -0,4617 | 7,24E-05 |
| rs79529740 | 12 | A | G | G | 0,212 | 0,061 | SLCO1B3 | -0,9668 | 7,58E-05 |
| rs2026023 | 20 | A | G | G | 0,86 | 0,266 | CDH4 | 0,5005 | 7,64E-05 |
| rs7571700 | 2 | G | A | A | 0,339 | 0,116 | LOC105373524 | 0,7046 | 7,97E-05 |
| rs9526299 | 13 | G | C | C | 0,399 | 0,083 | LOC105370195 | -0,7844 | 8,09E-05 |
| rs74196373 | 10 | A | C | C | 0,514 | 0,489 | RSU1P1 | -0,43 | 8,09E-05 |
| rs7503232 | 17 | A | G | G | 0,247 | 0,462 | LOC107985081 | -0,4392 | 8,34E-05 |
| rs4415205 | 7 | G | A | A | 0,809 | 0,172 | 7q11.22 | -0,559 | 8,43E-05 |
| rs2031470 | 1 | G | A | A | 0,498 | 0,288 | 1q25.2 | -0,4708 | 8,62E-05 |
| rs13256463 | 8 | G | A | A | 0,0364 | 0,264 | LOC401478 | -0,4757 | 8,83E-05 |
| rs10869692 | 9 | A | G | G | 0,0316 | 0,298 | PCSK5 | 0,4891 | 9,36E-05 |
| rs806303 | 13 | G | A | A | 0,694 | 0,499 | DLEU1 | -0,427 | 9,49E-05 |
| rs11185381 | 1 | A | G | G | 0,126 | 0,183 | 1p21.1 | 0,5722 | 9,51E-05 |
| rs9275653 | 6 | A | G | G | 0,498 | 0,38 | LOC102725019 | 0,4465 | 9,69E-05 |

**Table S2.** SNPs whose interaction with rs697221 SNP were associated with survival trait with p-value not exceeding 9.97E-05. Notations: “rsid” is SNP name; “chr” denotes chromosome number; “EA” is an effective allele; “AA” is an alternative allele; “MA” denotes minor allele; “pS” denotes p-value in separate genetic analysis of association of a given SNP with survival trait; “MAF” is a minor allele frequency; gene is used for gene name; “b12” is a beta coefficient in logistic regression model characterizing association of the interaction of the rs697221 SNP with a SNP in the “rsid” column with survival trait; “p12” is the p-value of association between interaction of the rs697221 SNP with a SNP shown in the “rsid” column.

| **rsid** | **chr** | **EA** | **AA** | **MA** | **pS** | **MAF** | **gene** | **b12** | **p12** |
| --- | --- | --- | --- | --- | --- | --- | --- | --- | --- |
| **rs7926726** | 11 | A | G | G | 0,471 | 0.27 | 11p11.2 | 0.43 | 2.92E-07 |
| **rs10439180** | 2 | A | G | G | 0,147 | 0.44 | LRP1B | 0.3487 | 4.98E-06 |
| **rs36053120** | 15 | G | C | C | 0,249 | 0.07 | LINC01578 | -0,678 | 6,91E-06 |
| **rs4772715** | 13 | C | A | A | 0,212 | 0.27 | 13q33.2 | 0,3772 | 8,29E-06 |
| **rs4867045** | 5 | G | A | A | 0,107 | 0.32 | 5p13.3 | -0,36 | 9,11E-06 |
| **rs77920110** | 13 | A | G | G | 0,469 | 0.05 | 13q33.2 | -0,7799 | 1,01E-05 |
| **rs2853552** | 7 | G | A | A | 0,359 | 0.28 | HDAC9 | 0,3628 | 1,75E-05 |
| **rs3773650** | 3 | C | A | A | 0,993 | 0.19 | TGFBR2 | -0,3919 | 2,20E-05 |
| **rs7494834** | 15 | G | A | A | 0,00236 | 0.35 | DET1 | -0,3379 | 2,36E-05 |
| **rs724410** | 7 | A | C | C | 0,663 | 0.09 | 7q36.3 | 0,5759 | 2,37E-05 |
| **rs62534655** | 9 | C | A | A | 0,721 | 0.32 | PTPRD | 0,3428 | 2,39E-05 |
| **rs2143863** | 20 | A | G | G | 0,477 | 0.14 | SNRPB | -0,4587 | 2,64E-05 |
| **rs1081025** | 6 | C | A | A | 0,719 | 0.05 | 6q16.2 | 0,7313 | 3,24E-05 |
| **rs9615752** | 22 | C | A | A | 0,114 | 0.16 | 22q13.31 | -0,4252 | 3,60E-05 |
| **rs10987235** | 9 | G | A | A | 0,205 | 0.20 | 9q33.3 | -0,3823 | 4,21E-05 |
| **rs76928216** | 12 | A | G | G | 0,379 | 0.06 | LOC105369608 | 0,677 | 4,67E-05 |
| **rs117388418** | 20 | C | G | G | 0,491 | 0.06 | SLCO4A1 | -0,6526 | 4,86E-05 |
| **rs6430764** | 2 | A | G | G | 0,334 | 0.40 | HNMT | 0,3146 | 4,99E-05 |
| **rs13042637** | 20 | A | G | G | 0,184 | 0.28 | NDUFAF5 | 0,3533 | 5,19E-05 |
| **rs13152024** | 4 | G | A | A | 0,516 | 0.27 | SLC10A7 | -0,3406 | 5,26E-05 |
| **rs57355402** | 6 | A | G | G | 0,0345 | 0.11 | DNAH8 | 0,5031 | 6,07E-05 |
| **rs28604945** | 5 | T | A | A | 0,00717 | 0.13 | LOC105377750 | 0,4467 | 6,29E-05 |
| **rs4528714** | 2 | C | A | A | 0,702 | 0.19 | SH3BP4 | -0,3796 | 6,41E-05 |
| **rs34029367** | 7 | G | A | A | 0,172 | 0.22 | ZNF804B | 0,3694 | 6,80E-05 |
| **rs526820** | 19 | A | T | T | 0,622 | 0.48 | COX7A1 | 0,3013 | 6,91E-05 |
| **rs1125469** | 9 | A | G | G | 0,495 | 0.48 | PRUNE2 | 0,3019 | 7,03E-05 |
| **rs2333990** | 17 | G | A | A | 0,65 | 0.19 | RPTOR | -0,3921 | 7,23E-05 |
| **rs17434150** | 6 | C | A | A | 0,885 | 0.07 | ZFAND3 | -0,5897 | 7,24E-05 |
| **rs17381941** | 2 | A | C | C | 0,409 | 0.15 | LOC107985845 | -0,4121 | 7,40E-05 |
| **rs6069746** | 20 | A | G | G | 0,579 | 0.24 | CASS4 | -0,3476 | 7,72E-05 |
| **rs74144434** | 1 | A | G | G | 0,213 | 0.19 | ELK4 | -0,3715 | 7,78E-05 |
| **rs1869288** | 2 | A | G | G | 0,389 | 0.12 | 2p25.1 | -0,447 | 7,91E-05 |
| **rs12327122** | 18 | G | A | A | 0,343 | 0.42 | 18q23 | 0,3071 | 7,97E-05 |
| **rs3798920** | 6 | A | G | G | 0,254 | 0,085 | AGPAT4 | 0,5404 | 7,99E-05 |
| **rs11804672** | 1 | G | A | A | 0,144 | 0,175 | 1p36.12 | -0,3783 | 8,46E-05 |
| **rs558059** | 21 | C | A | A | 0,414 | 0,316 | TIAM1 | 0,3206 | 8,54E-05 |
| **rs12464157** | 2 | G | A | A | 0,013 | 0,086 | CCDC141 | -0,529 | 8,54E-05 |
| **rs10446831** | 4 | G | A | A | 0,0313 | 0,131 | LOC105377567 | -0,4523 | 8,88E-05 |
| **rs10265230** | 7 | G | A | A | 0,231 | 0,181 | 7q31.31 | -0,376 | 8,90E-05 |
| **rs12989816** | 2 | A | G | G | 0,605 | 0,218 | 2p22.3 | -0,3579 | 8,99E-05 |
| **rs72963225** | 18 | A | G | G | 0,31 | 0,154 | 18q12.1 | 0,402 | 9,03E-05 |
| **rs13044092** | 20 | G | A | A | 0,83 | 0,192 | 20p12.1 | 0,3765 | 9,21E-05 |
| **rs34548235** | 3 | A | C | C | 0,386 | 0,118 | STXBP5L | -0,4615 | 9,41E-05 |
| **rs11590511** | 1 | A | C | C | 0,806 | 0,096 | 1q32.1 | -0,5112 | 9,68E-05 |
| **rs638284** | 18 | A | G | G | 0,879 | 0,077 | 18p11.22 | 0,581 | 9,73E-05 |
| **rs1543591** | 10 | A | G | G | 0,404 | 0,447 | 10q26.2 | 0,2843 | 9,76E-05 |
| **rs34294418** | 3 | T | A | A | 0,897 | 0,14 | LINC02026 | 0,4426 | 9,97E-05 |
